# Supplementary material for: Bleaching of Idesia polycarpa Maxim. Oil Using a Metal-Organic Framework-Based Adsorbent: Kinetics and Adsorption Isotherms
Source: Foods. 2025 Feb 25;14(5):787. doi: 10.3390/foods14050787 (PMC11898808; doi:10.3390/foods14050787)
Supplement: Supplementary file 1 [file foods-14-00787-s001.zip › foods-3450636-supplementary.pdf]

Supplementary materials

Bleaching of *Idesia polycarpa* Maxim. Oil Using a Metal-Organic Framework-Based Adsorbent: Kinetics and Adsorption Isotherms

Supplementary Table S1 Parameters of pseudo-first-order, pseudo-second-order, and intraparticle diffusion kinetic equations for carotenoid and chlorophyll.

Model	Kinetic parameter		Carotenoid	Chlorophyll
Pseudo-first-order	k_1		0.0486	0.0079
	q_e		14.16	0.3235
	R^2		0.9907	0.9721
Pseudo-second-order	k_2		0.0029	0.0081
	q_e		17.57	0.562
	R^2		0.9777	0.9721
Intraparticle diffusion	Phase I	k_1	1.6865	0.0122
		C_1	0	-0.0006
		R^2	1	0.9740
	Phase II	k_2	2.3386	0.0251
		C_2	-2.128	-0.0725
		R^2	0.9665	0.8904
	Phase III	k_3	0.2413	/
		C_3	11.45	
		R^2	0.9037	

Supplementary Table S2 Fitting parameters for carotenoid and chlorophyll adsorption isotherms.

Pigment	Model	Parameter	Value	R^2
Carotenoid	Langmuir	K_L	4.006E-7	0.9604
		q_m	1012	
	Freundlich	K_F	0.2151	0.9766
		n	1.128	
	Temkin	A_T	-242.4	0.9537
		b_T	60.74	
	Toth	Q_{Toth}	1012	0.9604
		K_{Toth}	4.006E-4	
		n_{Toth}	58.04	
Chlorophyll	Langmuir	K_L	7.24E-4	0.7655
		q_m	0.0221	
	Freundlich	K_F	0.0249	0.9480
		n	0.7147	
	Temkin	A_T	-0.0045	0.9482
		b_T	0.0518	
	Toth	Q_{Toth}	0.0221	0.9470
		K_{Toth}	0.5340	
		n_{Toth}	-1.060	

Supplementary Table S3 Thermodynamic parameters of MIL-88B(Fe) adsorption of carotenoid and chlorophyll.

Temperature (K)	Carotenoid			Chlorophyll		
	ΔG^0 (kJ mol^{-1})	ΔH^0 (kJ mol^{-1})	ΔS^0 (J mol^{-1} K^{-1})	ΔG^0 (kJ mol^{-1})	ΔH^0 (kJ mol^{-1})	ΔS^0 (J mol^{-1} K^{-1})
353	-12.14			-4.994		
363	-13.36			-5.968		
373	-16.90	64.00	0.1578	-8.658	45.34	0.0851
383	-19.30			-9.135		
393	-19.87			-10.50		
